# Virtual Reality Interventions in Obesity and Eating Disorders: A Systematic Review of Biomarker Outcomes

**DOI:** 10.1192/bjo.2026.11203

**Published:** 2026-06-30

**Authors:** Wafa Abdulrahman Alharbi, Hubertus Himmerich, Janet Treasure

**Affiliations:** 1 King’s College London, London, United Kingdom; 2 Taibah University, Madinah, Saudi Arabia

## Abstract

**Aims:**

Obesity and eating disorders (EDs) are associated with high morbidity, mortality, and limited long-term treatment efficacy. Virtual reality (VR) interventions are increasingly used in these conditions, yet their effects on objective biomarker outcomes have not been systematically evaluated. This review aimed to synthesize and critically appraise evidence on the effects of immersive VR interventions on clinical and biomarker outcomes in obesity and EDs.

**Methods:**

A systematic review was conducted following PRISMA 2020 guidelines. PubMed, Web of Science, and PsycINFO were searched from inception to April 2025. Eligible studies evaluated immersive VR interventions targeting obesity or EDs and reported at least one biomarker outcome, including anthropometric, physiological/autonomic, endocrine/metabolic, or neurocognitive measures. Randomized controlled trials (RCTs), non-randomized trials, and observational studies were included. Risk of bias was assessed using Joanna Briggs Institute tools. Random-effects meta-analyses were conducted when three or more RCTs reported comparable outcomes; otherwise, a structured narrative synthesis was conducted.

**Results:**

Twenty-three studies (N=1,413; obesity=11, EDs=12) were included. ED populations comprised individuals with anorexia nervosa, bulimia nervosa, and binge-eating disorder.

Obesity: VR-enhanced cognitive–behavioural therapy (VR-CBT) promoted sustained weight loss, whereas VR-based exercise interventions produced short-term reductions in BMI and body weight. Meta-analysis of four RCTs revealed a significant pooled effect of VR versus control on anthropometric outcomes (Hedges’ g=−0.35, 95% CI [−0.61,−0.09], p=0.009; I²=0%), with stronger effects for VR-CBT. Long-term follow-up (6–12 months) confirmed sustained benefits (g=−0.39, 95% CI [−0.73,−0.05]). Within-group pre–post analyses consistently showed reductions in BMI and weight (g=−0.56).

Eating Disorders: VR-CBT consistently reduced body image disturbance and fear of weight gain, while VR cue-exposure therapy decreased binge-eating and purging behaviours. Eye-tracking measures revealed attentional biases toward disorder-relevant cues. Autonomic markers (heart rate, heart rate variability, skin conductance), endocrine biomarkers (cortisol, α-amylase, leptin), and enzymatic measures confirmed that VR reliably elicited measurable psychophysiological responses.

**Conclusion:**

Immersive VR interventions show promise for improving clinical outcomes and eliciting measurable biomarker changes in obesity and EDs. Evidence supports VR-CBT for sustained anthropometric improvements and highlights VR’s potential for investigating autonomic and neurocognitive mechanisms. Larger, biomarker-integrated RCTs are needed to confirm efficacy and inform personalized, mechanism-driven treatment approaches.

